# Supplementary figures and images for: Left-dominance for resting-state temporal low-gamma power in children with impaired word-decoding and without comorbid ADHD
Source: PLoS One. 2023 Dec 29;18(12):e0292330. doi: 10.1371/journal.pone.0292330 (PMC10756518; doi:10.1371/journal.pone.0292330)

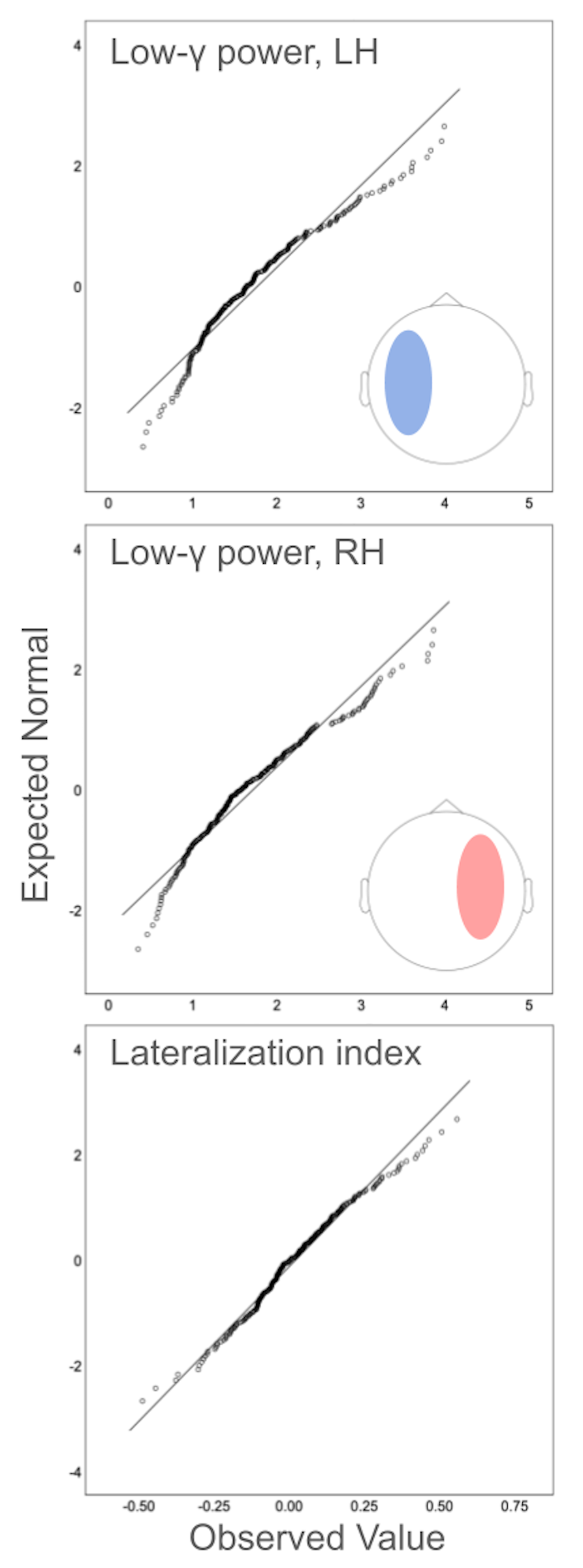

Supplement: S1 Fig — Presented data is after exclusion of outliers. Observed values for all variable distributions are compared to an expected normal distribution. More data points which fall along the diagonal indicate greater normality. (TIF) [file pone.0292330.s001.tif]

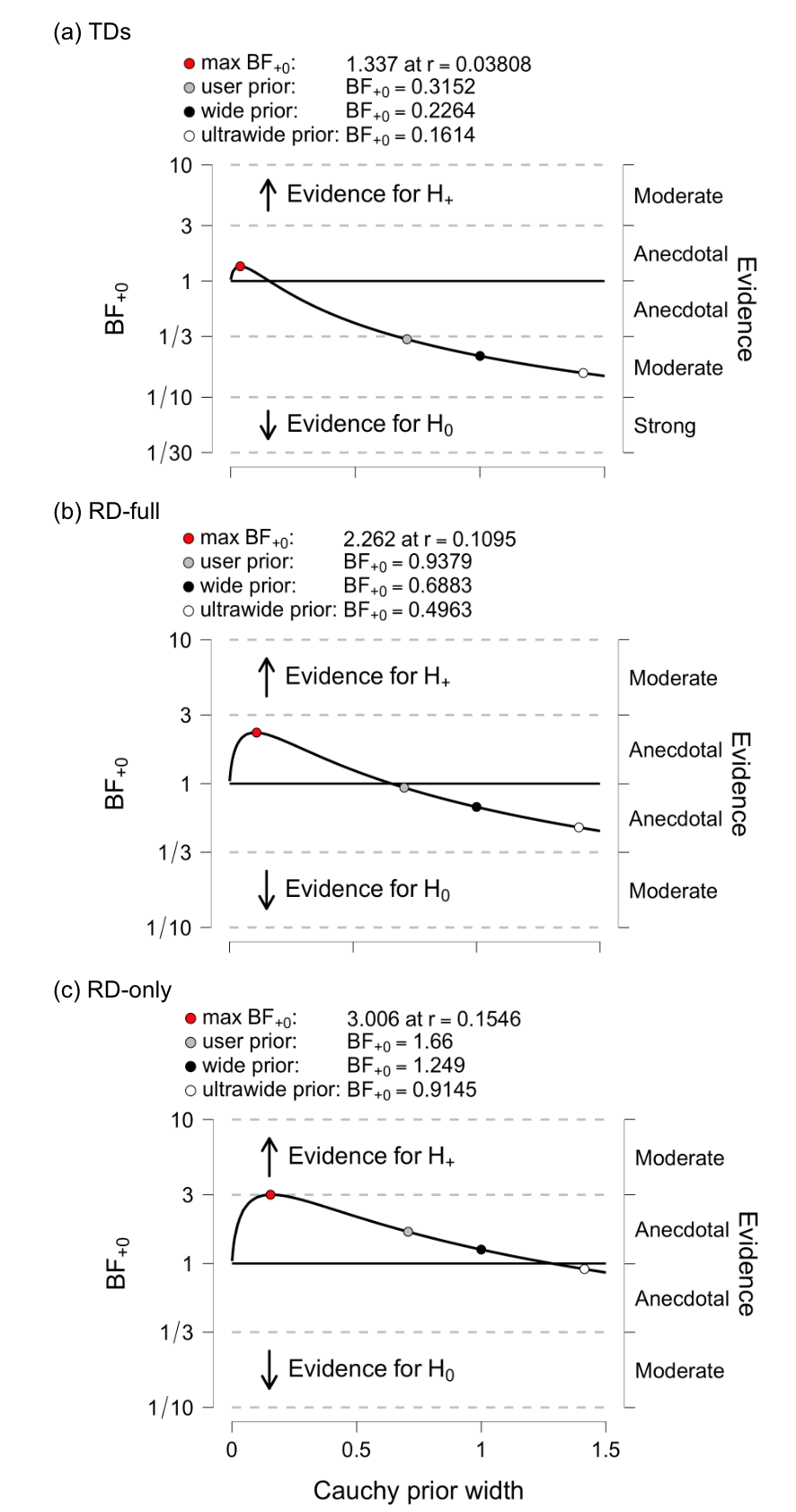

Supplement: S2 Fig — Plots for each group t-test show how sensitive the Bayes factor is to changes in the initial Cauchy prior width (X-axis). Y-axis indicates the value of the Bayes factor with the given prior; the ordinal scale on the right (‘Evidence’) is a colloquial interpretation for the corresponding y-value. Reported results in the main text are derived from the default user prior (gray dot). (a) = TRs; (b) = RD-full; (c) = RD-only subgroup. (TIF) [file pone.0292330.s002.tif]

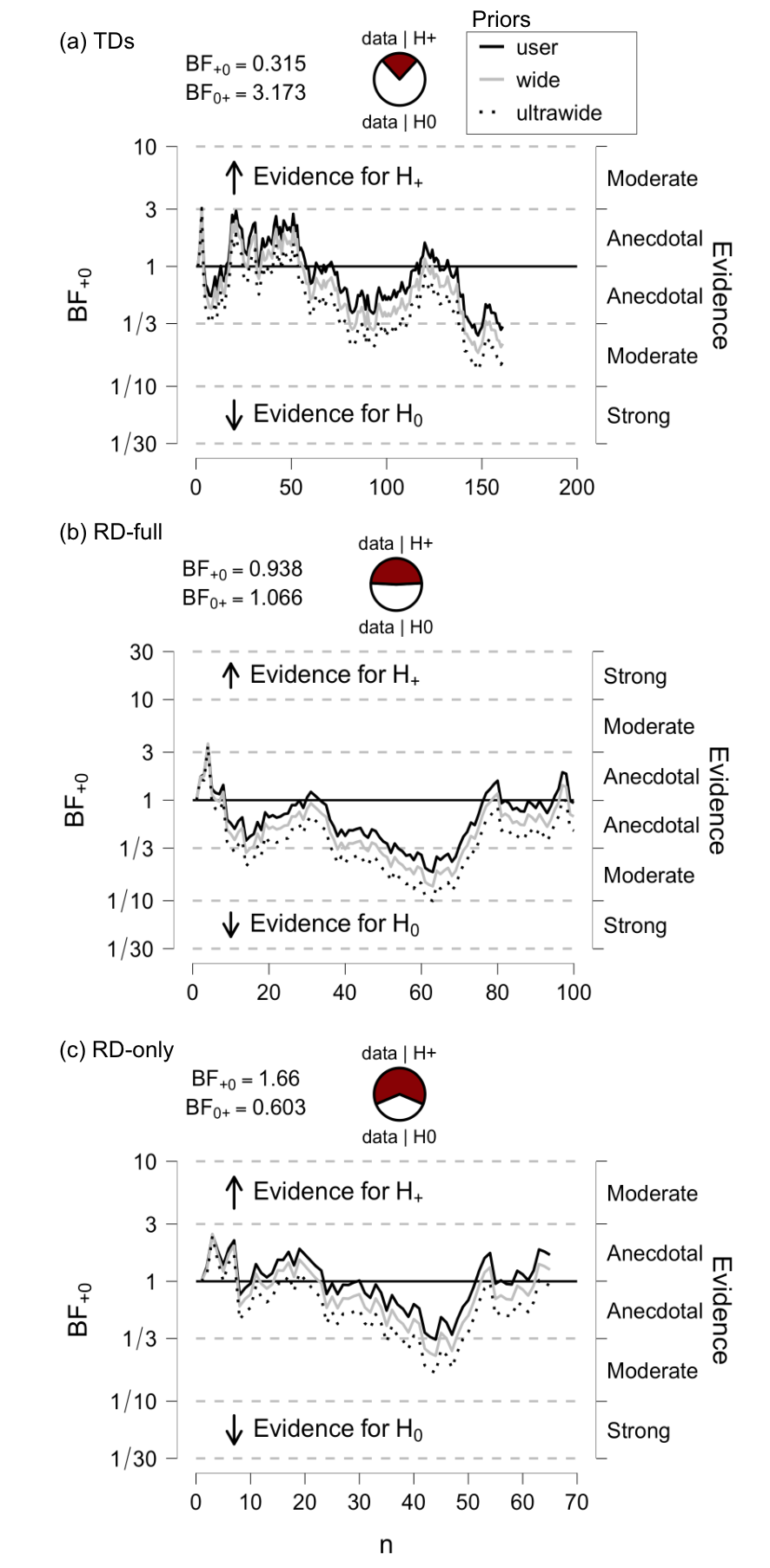

Supplement: S3 Fig — These tests indicate how the quantified degree of evidence changes as additional ‘samples’ are added. X-axis (n) indicates sample size. Y-axis reflects the value of Bayes factors at the given sample size; the ordinal scale on the right (‘Evidence’) is a colloquial interpretation for the corresponding y-value. Line style indicates the value of the Cauchy prior. (a) = TRs; (b) = RD-full; (c) = RD-only subgroup. (TIF) [file pone.0292330.s003.tif]
