# Supplementary material for: Left-dominance for resting-state temporal low-gamma power in children with impaired word-decoding and without comorbid ADHD
Source: PLoS One. 2023 Dec 29;18(12):e0292330. doi: 10.1371/journal.pone.0292330 (PMC10756518; doi:10.1371/journal.pone.0292330)
Supplement: S1 Table — SES, socioeconomic status (annual income). All p-values are two-sided. Age is given in years, with MEAN (SD) format. SES is given as median. Sex and dominant hand are percentages. Frequentist tests significant at the .05 α-level and Bayesian t-tests with a BF10 > 2.00 [equivalent to a log(BF10) > .70] are bolded. Hand dominance was determined using the Grooved Pegboard Test. The only significant contrast for demographic variables occurred for sex, such that the participants with anxiety had a higher proportion of girls than the participants without anxiety. All comparisons for low-gamma and the lateralization index favored the null hypothesis with a relative likelihood >2.00 [log(BF10) < -.70]. (DOCX) [file pone.0292330.s004.docx]

| Anxiety disorders | | | | Language disorder | | | |
| --- | --- | --- | --- | --- | --- | --- | --- |
|  | **–**  **(N = 218)** | **+**  **(N = 43)** |  |  | **–**  **(N = 239)** | **+**  **(N = 22)** |  |
| Age (yrs.) | 9.3 (1.8) | 9.2 (1.7) | t(259) = .55 (.581) | Age (yrs.) | 9.3 (1.8) | 9.1 (1.9) | t(259) = .48 (.629) |
| Sex (% fem.) | 43.6 | 62.8 | **X^2^(1) = 5.33 (.021)** | Sex (% fem.) | 47.7 | 36.4 | X^2^(1) = 1.04 (.308) |
| SES | 9.00 | 10.00 | Mann–Whitney U = 4085.50 (.173) | SES | 10.00 | 8.00 | Mann–Whitney U = 2818.00 (.568) |
| Dom. hand (% right) | 88.5 | 88.4 | X^2^(1) = .00 (.976) | Dom. hand (% right) | 87.9 | 95.5 | X^2^(1) = 1.14 (.286) |
| *Low-gamma differences due to Dx* | | | **log(BF_10_)** | ***Low-gamma differences due to Dx*** | | | **log(BF_10_)** |
| Low-gamma power, LH | | | -.71  δ = .227  [-.086, .546] | Low-gamma power, LH | | | -1.43  δ = -.050  [-.458, .354] |
| Low-gamma power, RH | | | -.86  δ = .209  [-.104, .527] | Low-gamma power, RH | | | -1.30  δ = -.117  [-.529, .287] |
| Lateralization index | | | -1.68  δ = .045  [-.266, .359] | Lateralization index | | | -1.23  δ = .139  [-.265, .552] |
