# Supplementary material for: Left-dominance for resting-state temporal low-gamma power in children with impaired word-decoding and without comorbid ADHD
Source: PLoS One. 2023 Dec 29;18(12):e0292330. doi: 10.1371/journal.pone.0292330 (PMC10756518; doi:10.1371/journal.pone.0292330)
Supplement: S2 Table — SES, socioeconomic status (annual income). All p-values are two-sided. Age is given in years, with MEAN (SD) format. SES is given as median. Sex and dominant hand are percentages. Frequentist tests significant at the .05 α-level and Bayesian t-tests with a BF10 > 2.00 [equivalent to a log(BF10) > .70] are bolded. Hand dominance was determined using the Grooved Pegboard Test. In typical readers, the only significant contrast for demographic variables occurred for sex, such that the participants with anxiety had a higher proportion of girls than the participants without anxiety. All comparisons for low-gamma and the lateralization index favored the null hypothesis with a relative likelihood >1.78 [log(BF10) < -.58]. (DOCX) [file pone.0292330.s005.docx]

| Anxiety disorders | | | | Language disorder | | | |
| --- | --- | --- | --- | --- | --- | --- | --- |
|  | **–**  **(N = 128)** | **+**  **(N = 33)** |  |  | **–**  **(N = 153)** | **+**  **(N = 8)** |  |
| Age (yrs.) | 9.2 (1.8) | 9.3 (1.7) | t(159) = -.34 (.735) | Age (yrs.) | 9.2 (1.8) | 8.5 (2.0) | t(159) = 1.18 (.241) |
| Sex (% fem.) | 43.0 | 69.7 | **X^2^(1) = 7.50 (.006)** | Sex (% fem.) | 49.0 | 37.5 | X^2^(1) = .40 (.525) |
| SES | 10.00 | 11.00 | Mann–Whitney U = 2021.00 (.689) | SES | 10.00 | 11.00 | Mann–Whitney U = 531.00 (.509) |
| Dom. hand (% right) | 89.1 | 87.9 | X^2^(1) = .04 (.847) | Dom. hand (% right) | 88.9 | 87.5 | X^2^(1) = .02 (.903) |
| *Low-gamma differences due to Dx* | | | **log(BF_10_)** | ***Low-gamma differences due to Dx*** | | | **log(BF_10_)** |
| Low-gamma power, LH | | | -1.04  δ = .191  [-.169, .559] | Low-gamma power, LH | | | -.58  δ = -.308  [-.976, .299] |
| Low-gamma power, RH | | | -1.17  δ = .166  [-.194, .532] | Low-gamma power, RH | | | -.60  δ = -.299  [-.966, .307] |
| Lateralization index | | | -1.50  δ = .070  [-.290, .433] | Lateralization index | | | -1.07  δ = -.019  [-.637, .595] |
