# Supplementary material for: Left-dominance for resting-state temporal low-gamma power in children with impaired word-decoding and without comorbid ADHD
Source: PLoS One. 2023 Dec 29;18(12):e0292330. doi: 10.1371/journal.pone.0292330 (PMC10756518; doi:10.1371/journal.pone.0292330)
Supplement: S4 Table — SES, socioeconomic status (annual income); PIQ, performance/nonverbal IQ; TOWRE, Test of word reading efficiency; PDE, phonemic decoding efficiency; SWE, sight-word efficiency. All p-values are two-sided. Cell values for age and PIQ are MEAN (SD). SES is given as median. RD, ADHD, sex, and handedness are percentages. Hand dominance was determined using the Grooved Pegboard Test. All group comparisons of demographic and behavioral variables between included vs. excluded (based on the minimum 100 ‘good’ epoch criterion) participants, including rates of RD/ADHD diagnoses, are n.s. at the .05 α-level. (DOCX) [file pone.0292330.s007.docx]

|  | Included (N = 262) | Excluded (N = 53) |  |
| --- | --- | --- | --- |
| Age, years | 9.3 (1.8) | 9.0 (1.8) | t(313) = 1.32 (.187) |
| Sex, % female | 46.9 | 45.3 | X^2^(1) = .05 (.825) |
| % RD | 38.5 | 41.5 | X^2^(1) = .16 (.687) |
| % ADHD | 13.4 | 15.1 | X^2^(1) = .11 (.737) |
| SES | 9.50 | 10.00 | Mann–Whitney U = 6928.50 (.980) |
| Dominant hand (% right) | 88.5 | 84.9 | X^2^(1) = .55 (.458) |
| PIQ, block design | 9.7 (3.0) | 9.7 (3.3) | t(313) = -.17 (.863) |
| *TOWRE scaled scores* | | | |
| PDE | 93.0 (21.0) | 94.8 (22.3) | t(313) = -.59 (.558) |
| SWE | 95.3 (22.3) | 96.5 (24.1) | t(313) = -.35 (.727) |
